# Identifying weak signals to prepare for uncertainty in the energy sector

**DOI:** 10.1016/j.heliyon.2023.e21295

**Published:** 2023-10-20

**Authors:** Nicholas Gilmore, Ilpo Koskinen, Patrick Burr, Edward Obbard, Alistair Sproul, Georgios Konstantinou, Jose Bilbao, Rahman Daiyan, Merlinde Kay, Richard Corkish, Iain Macgill, Emma Lovell, Chris Menictas, Anna Bruce

**Affiliations:** a School of Mechanical and Manufacturing Engineering, UNSW, Sydney, 2052, Australia; b School of Photovoltaic and Renewable Energy Engineering, UNSW, Sydney, 2052, Australia; c School of Electrical Engineering and Telecommunications, UNSW, Sydney, 2052, Australia; d School of Chemical Engineering, UNSW, Sydney, 2052, Australia

## Abstract

This study aims to prepare the energy sector for uncertainty using a foresight tool known as *weak signals.* Weak signals (subtle signs of emerging issues with significant impact potential) are often overlooked during strategic planning due to their inherent predictive uncertainty. However, the value does not lie in precise forecasting but in broadening the consideration of future possibilities. By proactively monitoring and addressing these otherwise neglected developments, stakeholders can gain early awareness of threats and opportunities and enhance their resilience, adaptability, and innovation.

A panel of technology experts identified eight weak signals in this study: 1) growing mistrust and local grid security measures, 2) consumer reactions to overly prescriptive policies, 3) long-term forecasting errors for thin-margin projects, 4) emergence of variable power industries, and 5) establishment of intercontinental transmission precedence; including three potential ‘wild cards’ requiring proactive mitigation: 6) escalating electrical generation dependence on continued imports, 7) a new threat surpassing climate change, and 8) mass deployment of low-emissions technology triggering a runaway loss of social license.

Political factors were the predominant source of uncertainty, as decisions can suddenly transform the energy landscape. Economic, technological, and social factors followed closely behind, generally through the emergence of new industries and behavioural responses. While environmental and legal factors were less frequent, stakeholders should still adopt a holistic approach, as the signals were found to be highly interconnected. Organisations should also assess their local context when applying these findings and continuously update and respond to their own list of weak signals.

## 1 Introduction

**Fig. 1:**
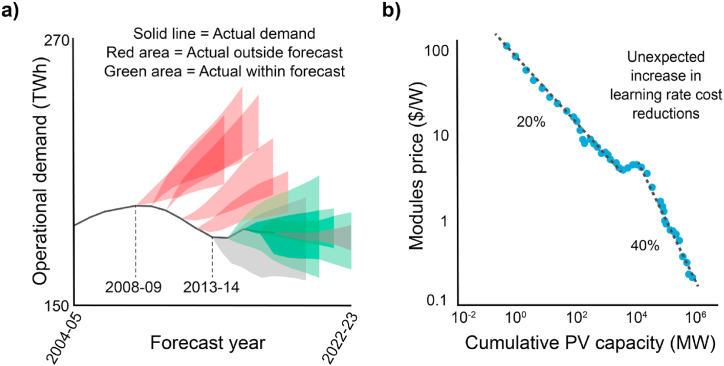
Historical example of energy forecast errors.

The future of energy is uncertain as technological advancements and geopolitical disruptions are challenging to anticipate. Amidst this uncertainty, leaders must still make strategic planning decisions with long-term ramifications. Faced with this challenge, many organisations over-rely on predictive forecasts leading to costly reactive interventions when they fail, as demonstrated in Fig. 1 (a, b).

Forecast models by the Australian Energy Market Operator have previously failed to predict future operational demand in the Australian National Electricity Market (a) resulting in the need for rapid and costly reactive interventions; graphic adapted from Ref. [1]. The International Energy Agency faced similar predictive challenges for photovoltaic module costs [2]. Serendipitous interactions between Australian, Chinese and American parties [3] partly caused the incorrect predictions associated with the trend-break of Wrights Law (b). Other trends have been broken by unexpected events such as a nuclear accident, global pandemic, and trade embargoes.

Conventional forecasts support optimal decision-making based on probabilistic and deterministic assumptions about future events. This approach is well suited to stable systems with high path dependency [4]. However, the efficacy of forecasts fades with increasing uncertainty [5] that may stem from 1) data quality and availability, 2) model accuracy and assumptions, 3) exposure to external factors and bias, 4) length of time horizon and 5) level of aggregation. In these situations, strategic foresight offers valuable tools as it assumes future events are uncertain a priori [6,7]. One well-known tool is scenario planning, which is increasingly used by private companies, national governments and multilateral institutions [6,8].

Scenario planning prepares organisations for multiple plausible futures by creating a set of narrative descriptions called scenarios. These scenarios are systematically developed in a set that includes meaningful differences with reference to driving forces and external contexts. By articulating future uncertainties in an accessible format, scenarios increase stakeholders' awareness of and engagement with potential changes and their implications. In turn, this enhances organisations' adaptability and resilience to unexpected changes [9,10] - Schwartz's ‘The Art of the Long View’ provides a more in-depth introduction for those unfamiliar with the field [11].

In our preceding foresight study, three *future* scenarios for energy were derived from an expert-led review of technologies in the Australian context [12,13]. The future scenarios encapsulated an array of potential changes in a simple and accessible format to facilitate prospective dialogue among diverse disciplines. However, the broad visions in this study did not systematically assess the strategic implications of the potential changes they aggregated. The current study employs a scenario planning tool called weak signals to address this gap.

Weak signals have many definitions, including early signs of possible but unconfirmed changes [14] and the first signs of emerging issues with characteristically low visibility [15]. Massé provides an instructive *germ* analogy “A sign which is slight in terms of present dimensions but huge in terms of its virtual consequences” [16]. In this study, weak signals are defined as early signs of impactful change that are difficult to predict and lie outside the awareness of stakeholders who control means to mitigate their impact [[17], [18], [19]]. This definition aims to clarify who observes the ‘low visibility’, ‘slight present dimension’, or ‘early sign’ and is further systemised in Section 2 Methods.

**Fig. 2:**
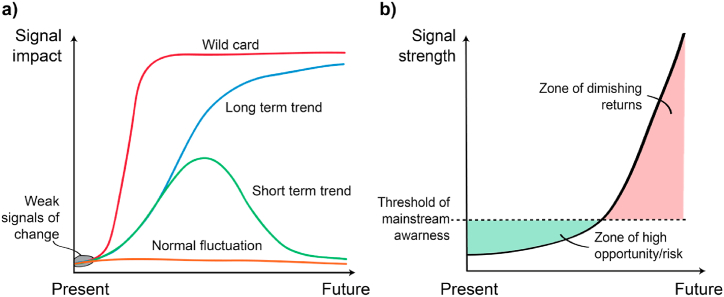
Potential impact of weak signals (a) Changes from weak signals may impact rapidly (wild card), gradually (long-term trend), fleetingly (short-term trends), or not at all (normal fluctuation) [23]. The COVID-19 pandemic and conflict in Ukraine provide wild card examples. (b) Acting on weak signals earlier presents a more significant opportunity/risk as mainstream awareness is low. Although awareness of signals likely exists within pockets of the community, more mainstream awareness is required for a significant planning response—figures adapted from Hiltunen [15].

Strategic plans should not neglect weak signals despite their low perceived individual likelihood, as their collective quantity and disproportionate impact potential means some will likely affect the future. Stakeholders may respond to weak signals in a hedged fashion, progressively committing resources to each signal until the certainty and relevance of the likely outcomes improve. This approach allows organisations more time to respond during the earliest stages when relative opportunities and risks are the most significant, Fig. 2 (a, b).

As weak signals represent early signs of change, most will not materialise. However, some will gradually form *trends*, and a limited few will rapidly manifest as *wild cards* (Fig. 2 left). Wild cards are sudden and fast-acting changes that are difficult to predict and may lead to significant disruption [[20], [21], [22], [23]]. To limit this disruption, wild cards often require a proactive, rather than a reactive, response, as systems usually lack the latent capacity to absorb their impacts. This study proposes the evaluation of ‘urgency of response’ to prioritise weak signals that may emerge as wild cards, building on the future practice by the UK Government Office for Science [24].

Monitoring weak signals allows organisations to anticipate threats and opportunities early, and previous studies have reported that integration into strategic planning processes may improve their robustness, flexibility and innovation [15,[25], [26], [27]]. A longitudinal study by Rohrbeck and Gemünden showed firms with vigilant foresight practices in 2008 were 33 % more profitable than the average and had 200 % additional capitalisation growth than the average [28] during 2015 [28]. These benefits were also supported by questionnaires of 408 companies across multiple industries in three independent studies [[29], [30], [31]] and another case study analysis of 20 applied foresight studies [32].

Despite evidence of strategic benefits, there is a distinct lack of work focused on identifying, interpreting, and representing weak signals in the literature. Dufva et al. [33], Flick et al. [34] and Bisson and Dinner [35] provide exemplary examples but do not target the energy sector. Retrospective studies on practised weak signal methods are more common [6,36,37], as are those on definitions [19]. Another cluster of work investigates data mining methods such as machine learning [14,38]. However, the efficacy of purely quantitative methods is limited as suitable databases and veritable correlations [39] constrain associated signals. These limitations support the established importance of involving qualitative expertise [14,40,41], further discussed in 2. Method.

This study addresses this critical gap in the literature by analysing weak signals for the energy sector based on a diverse panel of technology research experts. The research question investigates how the process of identifying, interpreting, and representing weak signals can enhance strategic planning practices. Crucially, the purpose of these weak signals is not to predict the future. Instead, they aim to encourage stakeholders to think more broadly about future possibilities. Weak signals highlight threats and opportunities at the earliest stages when they are often overlooked due to apparent uncertainty. Proactively addressing their possible impacts can enhance an organisation's resilience, adaptability, and innovation.

## 2 Method

### 2.1 Identification of signals

Signals were collected from two data sources: a *literature review* and an *expert panel.* These sources were selected as futurists have identified scientific researchers and journals as high-priority sources for weak signals of technology change [[40], [41], [42]]. Multiple source types are also preferred [14], and our proceeding *futures* study also included a comprehensive technology review which provides an initial list of priming signals for the current study [12,13].

### 2.2 Representation of signals

Each signal was represented by a short description of its potential change and impact. These descriptions were revised until the panel agreed they were clearly expressed and distinct. Although the panel deemed each signal distinct, some inextricable relationships remain, such as interdependencies from technological breakthroughs, geopolitical changes, or social and cultural shifts. Therefore, signals should be considered components of future change rather than mutually exclusive changes. Visual representations of the final list of signals were created using the DALL-E generative image model [43] to increase their accessibility.

### 2.3 Interpretation of signals

**Fig. 3:**
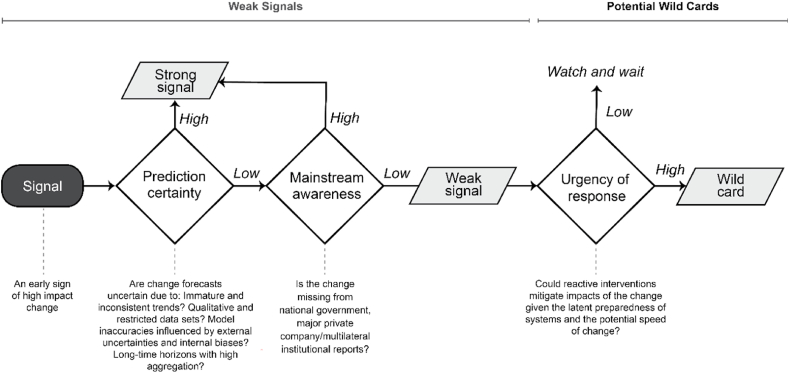
Process for identifying potential wild cards.

Each signal included a classification (high/low) and justification for prediction certainty, mainstream awareness, and response urgency. The certainty and awareness criteria are based on established definitions for strong and weak signals [14,15,17,18] and urgency from wild card definitions [18,[20], [21], [22], [23], [24]]. Each signal classification and justification were revised until panel consensus was reached. These classifications were then used to define each signal as strong, weak, or wild using the process described in Fig. 3. In practice, this process was highly iterative. It required robust discourse and many revisions to the descriptions and criteria justifications for each signal to achieve consensus. This time-intensive process ensured careful consideration of each signal, although it did limit the number of signals that could reasonably be considered, a previously reported challenge for foresight [44].

Signals of potentially impactful change were first collected from a literature review and expert panel, ensuring they were clear and distinct. They were then classified signals as a ‘Weak signal’ if panel consensus agrees it has a low prediction certainty and lacks mainstream awareness, and otherwise classified as a ‘Strong signal’. Lastly, weak signals were classified as ‘Wild cards’ if the panel agreed an urgent response is required to mitigate impacts, meaning the system is deemed unable to absorb fast-acting impacts with reactive interventions [5,45].

### 2.4 Validation of foresight quality

The quality of foresight depends on the *coverage* of possible futures and consideration of *causally relevant facts* [46] rather than predictive power [5]. To ensure adequate coverage, the 15 panellists (co-authors) were drawn from complementary fields of energy technology research [14] (solar, wind, nuclear, battery and hydrogen technologies). The maximum number of panellists was limited by the collaborative consensus process, which required a repeated and rigorous debate for each signal. However, this systematic approach also ensured careful consideration of causally relevant facts from expert knowledge and literature [46] – with panellists instructed to relate instructed to relate their scientific expertise with trends and drivers of changes reported in scientific and engineering literature to further limit the risk of conjecture.

### 2.5 Justification of qualitative data

The described method is based on the principles of foresight, which are fundamentally different from forecasting. While forecasts seek to predict the most likely future based on an extrapolation of current trends, foresight only aims to broaden understanding of potential futures, not to predict them [47].

Subjectivity remains integral to foresight as it seeks to explore a diverse range of possibilities, especially those that may be missed by purely data-driven approaches. This subjectivity may trigger a perceived lack of scientific rigour, often associated with qualitative methods. However, the validity and reliability of this study's data remain anchored in the panel's expertise, extracted through a rigorous process of consensus formation and further substantiated by literature.

This study acknowledges the validity and importance of quantitative planning methods. However, we posit they should be complemented, not replaced, by qualitative methods when planning for a sector as uncertain as energy – as it undergoes an unprecedented transition sensitive to a multiplicity of diverse factors. Here, the challenge is often not in collecting the data, which may be overabundant, but in interpreting its implications within a broader context. Interpretation is where the value of an expert panel and qualitative analysis becomes apparent, especially when substantiated by scientific literature where quantitative approaches are prevalent.

Furthermore, the advancement of quantitative methods does not negate the value of expert interpretation. Instead, the advent of new tools offers supplementary perspectives. However, these tools are not a replacement, as subtle and nuanced changes may require contextual understanding that is not easy to obtain through purely objective data and methods.

### 2.6 Replicability of signals

The signals presented by this study present one valid solution to the described method. However, replicating the method with a new panel of technology experts would likely produce different signals. This variability reflects the subjectivity of the approach, which is dependent on participants' knowledge, experience, and perspectives. However, these differences should not be viewed as a flaw as they would not undermine the purpose of either study. Instead, it reinforces the foresight assumption that multiple interpretations of future scenarios can broaden planning considerations.

Weak signals aim to promote and facilitate understanding of future developments. Lists of signals do not need to be exhaustive to encourage a continuous forward-thinking process, nor can they anticipate all possible changes, especially sudden or chaotic ones. However, the peripheral vision they do afford can enhance the resilience and adaptability of strategies, even if the considered changes are not all-encompassing or ultimately realised.

## 3 Weak signals of wild cards

This study identifies impactful changes that may emerge from (a) increasing dependence on consumable component imports, (b) displacement of the climate change threat, and (c) mass deployment depleting the social license of low-emissions technologies. These signals are considered wild cards, as proactive preparations are required to mitigate risks and seize opportunities.

### 3.1 Import dependence on consumable components

As their penetration increases, electricity generation and storage capacity increasingly depend on component imports for many countries with limited solar, wind and battery manufacturing. Australia provides an example, representative of many other states in Europe and North America. With only one 60 MW solar module production line in Australia, a disrupted supply chain would slow capacity growth from new installations and reduce operating capacity as old installations are decommissioned. Attrition from the latter would occur proportionately to the installation rate a lifetime ago, about 25 years [48], a risk exacerbated by Australia's world-leading deployment rate.

Some governments have addressed sovereign capabilities and critical minerals, although this risk of attrition by components is rarely identified. Proactive mitigations are required, as supply chain disruptions often outpace reactive measures. For example, manufacturing capabilities take years to develop. The *Solar Energy Manufacturing for America Act* aims to revive domestic solar manufacturing with tax credit incentives, which China's production prices have outcompeted since 2009. However, many nations are too small to support the scale and specificity required for electronic production. Other proactive responses may include stockpiling component reserves, expanding trade partnerships and diversifying the electricity mix.

### 3.2 Superseded climate change threat

The climate change threat emerged 50 years ago at the United Nations Scientific Conference in 1972. It has since replaced nuclear war as the predominant global threat in the public conscious, with comparatively few people campaigning for disarmament as a mass movement. This process could repeat itself. The COVID-19 pandemic temporarily curbed emissions [49], and the war in Ukraine replanned energy in Europe [50] – a more pervasive threat might emerge to displace climate change from a known (e.g. artificial intelligence [51] and biosecurity [52]) or unknown source. Failure to acknowledge this possibility could delay its detection and mitigation, undermining system preparedness for it.

Energy plans that optimise for emissions reductions at the detriment of resilience are exposed. These aggressive decarbonisation plans may become more common as increased warming becomes unavoidable. However, caution should be made when adjusting plans as the emergence of the threat is uncertain, and the slow global response to climate change suggests its threat is tenuously perceived [53].

### 3.3 Mass deployment depleting social license

Sheer material and space requirements may erode public acceptance, and constrain the widespread deployment of low emissions and low power density technologies to meet decarbonisation targets [54]. Land-use conflicts already impede solar and wind projects in several countries, and associated costs increase as suitable sites are used [55]. High penetration may also increase the frequency of social and ecological impacts, such as accumulating media reports of battery fire accidents and mounting concerns over electronic waste.

Adverse social reactions can escalate quickly. Nuclear power provides a stark historical example of a runaway loss of public acceptance. Media coverage of the Fukushima Daiichi accident led to an abrupt change in perceived risks, with policies phasing out existing nuclear generation in Germany and California, irrespective of the actual health and social impacts of the accident.

## 4 Weak signals of trends

Other impactful changes may emerge from (d) consumption rebound effects, (e) growing consumer mistrust, (f) overly prescriptive policies, (g) localized forecasting errors and (h) the setting of a new intercontinental electricity transmission precedence. However, these changes are generally considered weak signals, as the current level of preparations and the potentially slow rates of change means that proactive responses are not urgently required. However, monitoring of their potential impacts is warranted as this may change.

### 4.1 Mistrust and local grid security responses

Grid instability and spiking prices may erode consumer trust in central electricity suppliers, driving uptake of local grid security measures such as batteries and diesel generators, as shown by increased sales in Texas following multiple days of power disruptions. These local security issues present opportunities for those who provide microgrids and other local services, and risks for those who cannot access them.

Low population densities and long transmission distances have made fringe networks expensive to maintain for decades but, despite proposed plans, have yet to fragment networks into microgrids. This lack of change may be partly due to the entrenchment of the central grid, as mass integration of distributed resources is not straightforward. The uptake of distributed energy resources throttles the speed of this change. However, potential feedback effects from a rapid diffusion of local grid security measures should still be investigated to mitigate impacts.

### 4.2 Prescriptive energy policy reactions

High prices and grid instability create opportunities for prescriptive energy policies that may impact the adoption and use of distributed resources. Solar feed-in tariffs are a well-known example. However, Australia's leading uptake of rooftop solar has seen consumer data and control rights emerge as policy issues.

The *Consumer data right* means rooftop solar owners may only access their data via an accredited data recipient, with Energy Consumers Australia raising concerns over this process that was first developed for the banking sector [56]. Additionally, pilot studies often assume a central control of assets despite a lack of social science investigating consumer willingness to relinquish control. The Inverter Requirements (AS/NZS 4777.2) already allow the market operator to control home inverters. However, it is unclear whether the reactions of these early adopters can be extrapolated to the general population.

Despite growing attention, these policy issues are still poorly understood and may be complicated by emerging technologies such as digital twins. Proactive investigations are advisable, as social reactions to policy changes can occur rapidly. Besides South Australia, which regularly exceeds 100 % variable generation, Hawaii provides another forerunning case study for these issues [57].

### 4.3 Forecasting errors from local variations

Local weather will vary from the average of climate change. However, site forecasts for commercial generators rarely account for these localized variations. Solar irradiance is often collected over a year to forecast site feasibility. Temperature changes impact panel efficiencies and lifetimes. Panel efficiencies are also affected by aerosol particulates, dust during drought, and soot and humidity during bushfires. The soiling factors also affect maintenance and cleaning requirements, often contracted at the start of projects. For wind generators, pressure patterns may move over a decade investment period. Forecast models have coarse spatial resolutions and a limited understanding of atmospheric conditions [58], although neural networks may offer future accuracy improvements. Local land-use changes can also impact outputs, such as tree crop removal.

The most extreme variations will impact large projects with strict obligations and thin profit margins and may strand ancillary assets like transmission lines and substations. An economic incentive to accurately forecast site feasibility should lead to investments in improved practices as impacts are acknowledged. Although weather changes are gradual, projects with long investment periods may still be impacted.

### 4.4 Variable power industries

Industries that exploit abundant variable electricity might emerge. This possibility is addressed by several strategic reports [59] and acknowledged by private and public sector investment in hydrogen. Many data centres are curbing consumption through efficiency adaptations such as demand shifting and sector coupling. However, targeted policy interventions are required to improve understanding of associated rebound effects, as prediction methods are fragmented and unreliable [60]. Rebound effects can reduce actual versus potential energy savings from more efficient energy technologies, with most studies reporting effects between 0 % and 100 % [61,62]. However, this should not undermine the importance of energy efficiency, which is crucial for minimising increased consumption with economic growth [63]. Denmark encourages sufficiency adaptations through life cycle assessment requirements of the Danish Marketing Practices Act.

### 4.5 Intercontinental electricity precedence

Suncable plans to supply up to 15 % of land-limited Singapore's electricity from a massive, irradiant solar-battery site in Australia via a 4200 km submarine cable. The idea of a global power grid [64], energy super-powers [65] or energy from the desert [66], has been around for over a decade. However, success or failure could influence confidence in other intercontinental transmission projects, as the longest operational cable is the 720 km North Sea Link.

Inter-continental infrastructure is exposed to geopolitical relationships, natural disasters, massive capital requirements, environmental approvals, and novel technical problems. The severity of these challenges is reflected by the recent bankruptcy of the Nord Stream 2 pipeline. The concentration of resources has historically heightened the risk of conflict; decarbonisation may further increase the value of low-emissions power while transmission changes its geographic availability.

Infrastructure projects take years to plan, construct and commission. However, political reactions can occur more rapidly. Therefore, proactive preparations are required to mitigate risks, especially if the solution requires significant infrastructure development. Australia's 20-year Integrated System Plan is indicative of this kind of inflexibility.

## 5 Strong signals of trends

The following potential changes are considered strong signals, as governments and other large organisations are proactively preparing for the risks and opportunities. Each is described here, as this general level of preparation may vary depending on the audience's specific context.

### 5.1 Shifting critical mineral resource values

Critical minerals required for deploying new energy technologies may disrupt current resource values. These minerals are more geographically concentrated than traditional resources, shown historically to increase the risk of price volatility and conflict [67], and are exposed to geopolitical changes. Strained supplies may also push mines to regions vulnerable to environmental and social damage [68].

### 5.2 Hydrogen hype opportunity cost

Hydrogen has unprecedented political and business momentum, with at least 37 governments releasing or working on strategies [69]. However, the industry's future is hard to predict as it is relatively immature, encompasses many different technologies, and has risks to public acceptance, such as safety perceptions [70]. Overhyped expectations may cause an opportunity cost for competing technologies if hydrogen is portrayed to and perceived by the public as the solution to all energy problems.

### 5.3 Adaptive consumer behaviour

Distributed energy technologies allow new adaptive behaviours behind the meter. For example, well-insulated homes may be pre-cooled or pre-heated to exploit low solar electricity prices, and office workers may opt for unconventional hours to avoid electric vehicle charging during high-demand periods. The emergence of new consumer behaviours is difficult to predict, particularly given the dependence on new technologies and regulations. This uncertainty may expose central market operators that over-rely on consumer behaviours to balance the grid.

## 6 Discussion and conclusions

### 6.1 Strategic planning implications of weak signals

**Fig. 4:**
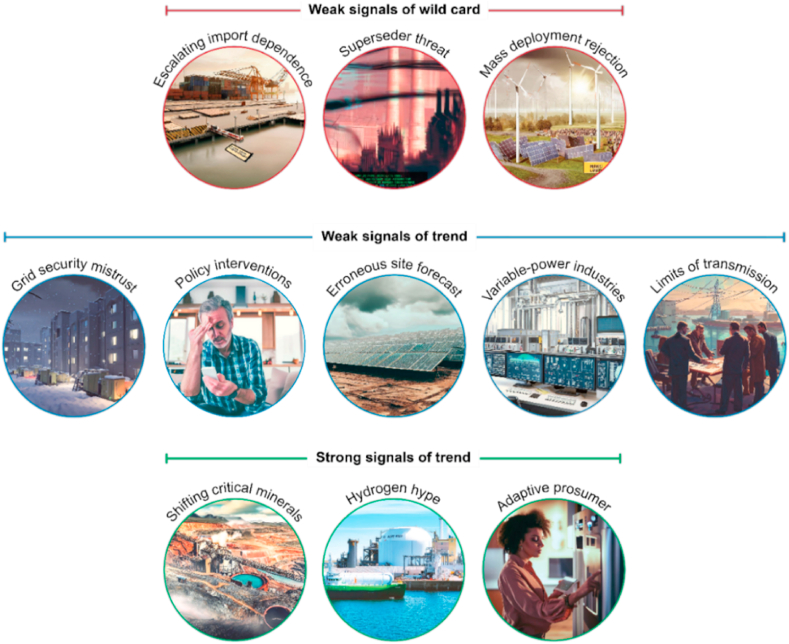
Graphical representations of signals identified in the current study generated by BING Image Creator powered by DALL-E.

In this foresight study, a panel of technology experts identified eight weak signals, which are early signs of high-impact change that are difficult to predict and lack mainstream awareness. These included: 1) local grid security measures in response to growing mistrust in central providers, 2) the implications of increasingly prescriptive distributed energy policies, 3) potential long-term forecasting errors for projects with thin margins, 4) new industries capitalising on abundant variable power, and 5) the establishment of intercontinental transmission precedents. Of these weak signals, three were deemed potential wild cards, fast-acting changes that require a proactive response to mitigate impacts and seize opportunities. These wild cards were 6) growing dependence of grid capacity on continuous component imports, 7) the possibility of climate change being superseded by another threat, and 8) mass deployment of low-emission technology triggering a runaway loss of social license. These are represented visually in Fig. 4.

Although these weak signals highlight potential opportunities and threats, their purpose is not to predict these outcomes. Instead, they aim to encourage stakeholders to consider broader possibilities by highlighting emerging changes often overlooked due to predictive uncertainty. In this way, they serve as an early warning system that enhances strategic plans’ resilience and adaptability while stimulating innovation. Even if these specific changes do not occur, the associated measures can prepare stakeholders for other changes, offering a way for organisations to prepare systematically for uncertainty.

For example, vulnerable component and material supply chains could be bolstered by increasing reserves, expanding trade partners, and developing sovereign manufacturing capabilities. Risks to social perceptions and behaviours could be mitigated by diversifying the energy mix, implementing public engagement programs, and promoting energy efficiency measures. Opportunities related to the increasing availability of variable power could be addressed through research and development of energy storage, demand management, and advanced production technologies. Lastly, the possibility of a threat that surpasses climate change in urgency and public consciousness could encourage planners to broaden international cooperation on security and optimise systems for a broader range of requirements such as economic productivity, ecological restoration and system resilience.

To apply the findings from this study, a thorough exploration of organisation-specific implications is required. As weak signals were identified by technology experts considering a global scale, organisations should analyse them for their region and sector. The organisation's weak signal process should also involve continuous monitoring and updating so that signals effectively catch emerging threats and opportunities. These actions can support organisational preparedness, although significant implementation challenges are presented by the complex interactions of political, economic, sociological, technological, environmental, and legal factors.

### 6.2 External factors for weak signals

Political factors were the predominant source of uncertainty for the weak signals in this study, as policy decisions can rapidly reshape the energy landscape. For example, this uncertainty may come from foreign trade and climate agreements, public infrastructure investment and market incentives, evolving security priorities, and new market standards and regulations. Given their significance, stakeholders should actively monitor political developments to plan for future uncertainty.

Economic, sociological and technological factors were also common sources of uncertainty, generally through the emergence of new industries and social responses, which are both difficult to predict. While environmental and legal factors were less common in the current weak signals, stakeholders should holistically consider external factors to avoid surprises, given their tangled interdependencies.

Each signal exhibited interdependencies, demonstrating the need for organisations to consider diverse factors when planning for future uncertainties. Sociological signals had the most connections, with a cluster regarding consumer behaviours and perceptions. Other clusters emerged around energy supply chains and new industries stemming from the growth of low-emission technologies.

### 6.3 Limitations and future work

The study has several limitations and areas of future work. Weak signals were sourced from technology experts. Expanding this to include social science, economics, and policy disciplines would provide a more holistic assessment. Furthermore, expert panellists were drawn from research institutions. The inclusion of other industries would make results more comprehensive. However, scientific and engineering researchers have been identified as a quality source of technological change and provided a reasonable scope for the present study. Additionally, integrating quantitative data may uncover potential changes imperceptible to this study's qualitative sources, with machine learning a promising method for overcoming existing limitations.

In this study, weak signals were based on a global scope. Before an organisation can apply these findings to their strategic plans, they should consider the implications of their local context. Furthermore, the signals represent a static list of potential changes developed during 2022. The relevance of these signals will diminish over time. Future work should consider efficient methods for monitoring and updating lists of signals and integrations into organisations' strategic planning processes.

## Data availability statement

Data will be made available on request.

## CRediT authorship contribution statement

**Nicholas Gilmore:** Conceptualization, Data curation, Formal analysis, Investigation, Methodology, Visualization, Writing – original draft, Writing – review & editing. **Ilpo Koskinen:** Conceptualization, Data curation, Formal analysis, Investigation, Methodology, Visualization, Writing – original draft, Writing – review & editing. **Patrick Burr:** Conceptualization, Data curation, Formal analysis, Investigation, Methodology, Writing – original draft, Writing – review & editing. **Edward Obbard:** Conceptualization, Data curation, Formal analysis, Investigation, Methodology, Writing – original draft, Writing – review & editing. **Alistair Sproul:** Conceptualization, Data curation, Formal analysis, Investigation, Methodology, Visualization, Writing – original draft, Writing – review & editing. **Georgios Konstantinou:** Conceptualization, Data curation, Formal analysis, Investigation, Methodology, Writing – original draft, Writing – review & editing. **Jose Bilbao:** Conceptualization, Data curation, Formal analysis, Investigation, Methodology, Visualization, Writing – original draft, Writing – review & editing. **Rahman Daiyan:** Conceptualization, Data curation, Formal analysis, Investigation, Methodology, Writing – original draft. **Merlinde Kay:** Data curation, Formal analysis, Investigation, Methodology, Writing – original draft, Writing – review & editing. **Richard Corkish:** Conceptualization, Data curation, Formal analysis, Investigation, Methodology, Writing – original draft, Writing – review & editing. **Iain Macgill:** Conceptualization, Data curation, Formal analysis, Investigation, Methodology, Writing – original draft, Writing – review & editing. **Emma Lovell:** Conceptualization, Data curation, Formal analysis, Investigation, Methodology, Writing – original draft, Writing – review & editing. **Chris Menictas:** Conceptualization, Data curation, Formal analysis, Investigation, Methodology, Writing – original draft, Writing – review & editing. **Anna Bruce:** Conceptualization, Data curation, Formal analysis, Investigation, Methodology.

## Declaration of competing interest

The authors declare the following financial interests/personal relationships which may be considered as potential competing interests:Richard Corkish acknowledges that this Program has been supported by the 10.13039/100015539Australian Government through the 10.13039/501100005105Australian Renewable Energy Agency (10.13039/501100005105ARENA).
